# Probiotic-related bacteremia after major hepatectomy for biliary cancer: a report of two cases

**DOI:** 10.1186/s40792-021-01216-5

**Published:** 2021-06-01

**Authors:** Mitsuhiro Shimura, Masamichi Mizuma, Kei Nakagawa, Shuichi Aoki, Takayuki Miura, Tatsuyuki Takadate, Kyohei Ariake, Shimpei Maeda, Kei Kawaguchi, Kunihiro Masuda, Masaharu Ishida, Hideo Ohtsuka, Takanori Morikawa, Takashi Kamei, Michiaki Unno

**Affiliations:** Department of Surgery, Tohoku University Graduate School of Medicine, 1-1 Seiryomachi, Aobaku, Sendai, 980-8574 Japan

**Keywords:** Probiotics, Bacteremia, Sepsis, Postoperative complication, *Clostridium butyricum*

## Abstract

**Background:**

Probiotics have been reported to be beneficial for the prevention of postoperative complications and are often used during the perioperative period. Among the probiotic-related adverse events, bacteremia is rare. Here, we report two cases of probiotic-related bacteremia after major hepatectomy for biliary cancer.

**Case presentation 1:**

A 74-year-old man was referred to our hospital to be treated for gallbladder cancer. Neoadjuvant chemotherapy, two courses of gemcitabine plus S-1 combination therapy, was administered. Extended right hepatectomy with caudate lobectomy, extrahepatic bile duct resection and biliary reconstruction were performed 3 weeks after chemotherapy. Probiotics, *Clostridium butyricum* (*C. butyricum*) MIYAIRI 588, were administered 6 days before surgery and continued after surgery. Sepsis of unknown origin occurred 17 days after surgery and developed into septic shock. *C. butyricum* was detected in blood cultures at postoperative day 26 and 45. After stopping the probiotic agent, *C. butyricum* was undetectable in the blood cultures. The patient died due to an uncontrollable sepsis 66 days after surgery.

**Case presentation 2:**

A 63-year-old man with diabetes mellitus whose past history included total colectomy, papillectomy, and Frey’s operation at the age of 19, 34 and 48, respectively, was referred to our hospital to be treated for perihilar cholangiocarcinoma. Extended left hepatectomy with caudate lobectomy, extrahepatic bile duct resection and reconstruction of bile duct were performed. Probiotics were administered during the perioperative period. Combined probiotics that included lactomin, amylolytic bacillus and *C. butyricum*, were given before surgery. *C. butyricum* MIYAIRI 588 was given after surgery. Sepsis occurred 16 days after surgery and developed to respiratory failure 8 days later. Blood culture at postoperative day 25 revealed *Enterococcus faecalis* and *C. butyricum*. After the probiotics were stopped at postoperative day 27, *C. butyricum* was not detected in the blood culture. The general condition improved with intensive care. The patient was transferred to another hospital for rehabilitation at postoperative day 156.

**Conclusion:**

It should be noted that the administration of probiotics in severe postoperative complications can lead to probiotic-related bacteremia.

## Background

Probiotics, defined as “Live microorganisms which when administered in adequate amounts confer a health benefit on the host” by the World Health Organization and Food and Agricultural Organization of the United Nations [1], are used in clinical settings worldwide for their protective effect against infection. In gastrointestinal surgery, probiotics have been reported to reduce postoperative infectious complications and are therefore widely administered during the perioperative period [2–6].

Probiotics are generally considered safe to use. However, a risk of probiotics-related infection, especially in patients with a poor immune condition, has been pointed out because probiotics are live microorganisms [7, 8]. Although probiotics-related bacteremia is rare, it can lead to fatal conditions [9]. Here, we present two cases of probiotics-related bacteremia that occurred during the treatment for sepsis after major hepatectomy with resection of extrahepatic bile duct for biliary cancer.

## Case presentations

### Case 1

**Fig. 1:**
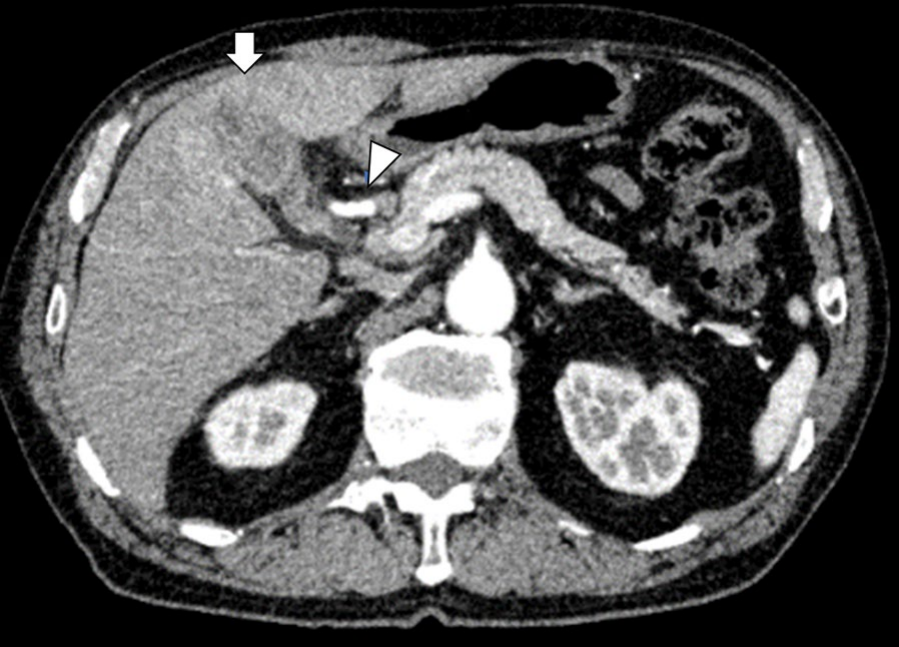
Preoperative enhanced CT imaging of case 1. The arrow indicates the main tumor. The arrowhead indicates the right hepatic artery (RHA). A tumor of the gallbladder infiltrated to the liver parenchyma and spread to the cystic and common hepatic duct with contact of RHA

**Fig. 2:**
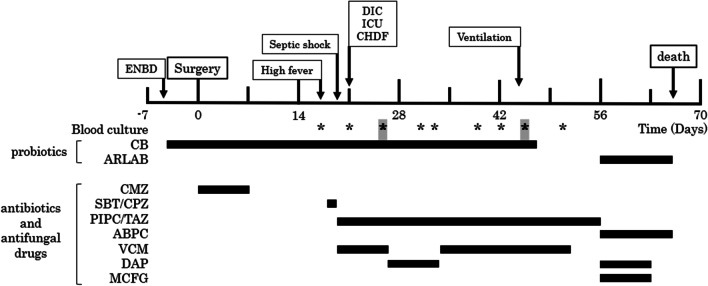
Postoperative course of case 1. Asterisks indicate blood culture tests. Bold asterisks highlighted in gray indicate positive blood culture of *Clostridium butyricum*. ABPC, ampicillin; ARLAB, antibiotic-resistant lactic acid bacteria; *C. butyricum*, *Clostridium butyricum*; CHDF, continuous hemodiafiltration; CMZ, cefmetazole; DAP, daptomycin; DIC, disseminated intravascular coagulation; EBD, endoscopic biliary drainage; ICU, intensive care unit; MCFG, micafungin; PIPC/TAZ, piperacillin/tazobactam; SBT/CPZ, sulbactam/cefoperazone; VCM, vancomycin hydrochloride

**Fig. 3:**
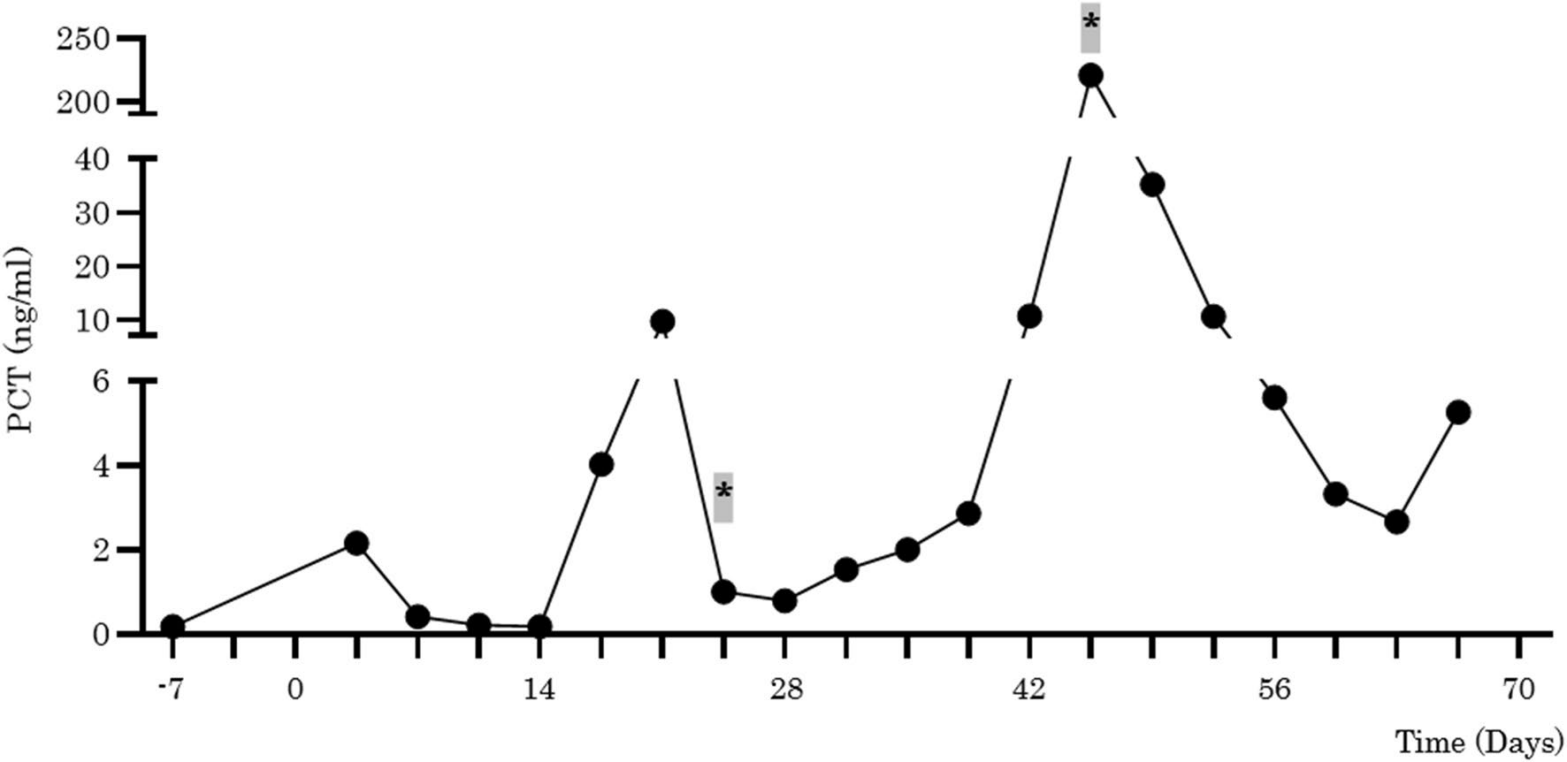
The change of serum procalcitonin value in case 1 during the perioperative period. A bold asterisk highlighted in gray indicates positive blood culture of *Clostridium butyricum*. PCT, procalcitonin

A 74-year-old man with hypertension visited a previous hospital due to upper abdominal pain, appetite loss and nausea for 3 months. The patient had a past history of sigmoid colon and prostate cancer at the age of 50 and 68, respectively. The patient was referred to our hospital for further examinations and treatment. Enhanced computed tomography (CT) scan revealed a tumor of the gallbladder with invasion to the liver parenchyma, spreading to the junction of the cystic duct, and in contact with the right hepatic artery (Fig. 1). The biopsy of the bifurcation of the hepatic duct histologically demonstrated adenocarcinoma. The patient was diagnosed as gallbladder cancer spreading to the perihilar bile duct. The patient received percutaneous transhepatic portal vein embolization for the right liver. After 1 week, neoadjuvant chemotherapy (NAC) with gemcitabine plus S-1 was started and was administered for 6 weeks. The patient underwent endoscopic biliary drainage (EBD) 1 week before surgery due to obstructive jaundice. Preoperative screening cultures of the nasopharynx and bile juice revealed no specific findings. Preoperative blood test showed liver disorder (AST 42 U/I, ALT 87 U/I), malnutrition (ALB 3.0 g/dl, pre-albumin 15.6 mg/dl, retinol binding protein (RBP) 2.4 mg/dl) and anemia (Hb 8.4 g/dl). C-reactive protein (CRP) was slightly elevated (0.6 mg/dl). Total cholesterol (T-Chol) was 154 mg/dl. The percentages of neutrophils and lymphocytes were 46.8% and 29.8%, respectively. Indocyanine green clearance of the predicted remnant liver (ICG-Krem) was 0.052. Extended right hepatectomy with caudate lobectomy, extrahepatic bile duct resection and biliary reconstruction were performed 3 weeks after NAC. Operating time was 464 min, and operative blood loss was 865 ml. According to the TNM classification of malignant tumors in the eighth edition of the Union for International Cancer Control (UICC), histological examination demonstrated ypT3N1M0 and R0. Figure 2 shows the perioperative clinical course. Probiotics *Clostridium butyricum* (*C. butyricum*) MIYAIRI 588 were started from 6 days before hepatectomy. The postoperative course went well without bile or anastomotic leakage until postoperative day 16. The patient had a high fever of 40 °C with *Enterococcus faecium* and *Enterobacter cloacae* positive in two independent blood cultures on postoperative day 17 (Table 1), progressing to septic shock on postoperative day 20. The origin of the infection was unknown. Antibiotics and antifungal drugs were administered according to the results of the drug sensitivity test (Fig. 2). Continuous hemodiafiltration was started from 21 days after surgery. In blood cultures at postoperative day 26, two of two sets were positive for *Enterococcus faecium*, while one of two sets was positive for *C. butyricum* (Table 1). Although *C. butyricum* might have been from the probiotics, we thought it was from contamination because *C. butyricum* was not positive in 2 sets of blood cultures. The serum procalcitonin (PCT) value was 1.0 ng/ml at postoperative day 26 (Fig. 3). Consequently, the probiotics continued to be administered. The next blood cultures detected no *C. butyricum*. Antibiotic-resistant lactic acid bacteria (ARLAB), *Enterococcus faecalis* 129 BIO 3B-R, were additionally administered from 29 days after surgery. *C. butyricum* was observed in the two sets of blood cultures 45 days after surgery, showing the extremely increased serum PCT value (221.0 ng/ml) (Fig. 3). Probiotic-related bacteremia derived from *C. butyricum* MIYAIRI 588 was strongly suspected. Therefore, it was stopped immediately after the results were obtained. In the next blood culture, *C. butyricum* was not detected. The patient eventually died on postoperative day 66 due to uncontrollable sepsis.

**Table 1:** Postoperative culture test of case 1

Collection date (postoperative day)	Sample	Detected bacteria
18	Blood	*Enterococcus faecium*^b^
		*Enterobacter cloacae*^b^
21	Blood	*Enterococcus faecium*^b^
		*Enterobacter cloacae*^a^
21	Sputum	Normal flora
		Coagulase (−) *Staphylococcus*
21	Stool	*Enterococcus* sp.
		*Citrobacter freundii*
		*Enterobacter cloacae*
		*Candida albicans*
26	Blood	*Enterococcus faecium*^b^
		*Clostridium butyricum*^a^
26	Sputum	Normal flora
		Coagulase (−) *Staphylococcus*
		*Enterococcus faecium*
		*Enterobacter cloacae*
32	Blood	Negative^c^
33	Blood	*Enterococcus faecium*^c^
36	Stool	*Enterococcus *sp.
		*Citrobacter freundii*
		*Enterobacter cloacae*
		*Candida albicans*
38	Blood	*Staphylococcus capitis*^b^
		*Enterococcus faecium*^a^
42	Blood	*Candida albicans*^b^
		*Enterococcus faecium*^a^
45	Blood	*Candida albicans*^b^
		*Clostridium butyricum*^b^
48	Sputum	*Candida albicans*
50	Blood	*Candida albicans*^c^

^a^Positive in one of two sets

^b^Positive in both of two sets

^c^One set examined

### Case 2

**Fig. 4:**
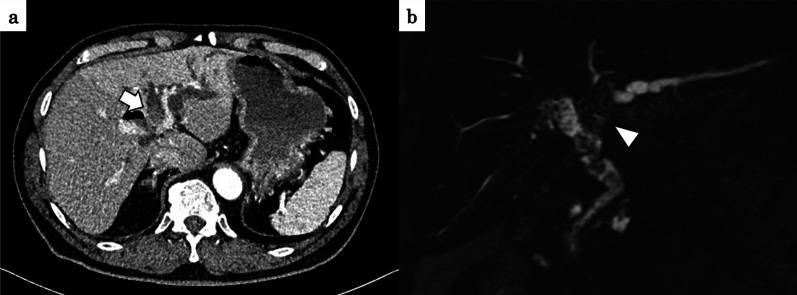
Preoperative imaging examinations of case 2. **a** Enhanced CT imaging showed tumor with a slight enhancement in the intrahepatic left bile ducts (white arrow). **b** Magnetic resonance cholangio-pancreatgraphy (MRCP) revealed a defect lesion from the common bile duct to the left bile duct (arrowhead)

**Fig. 5:**
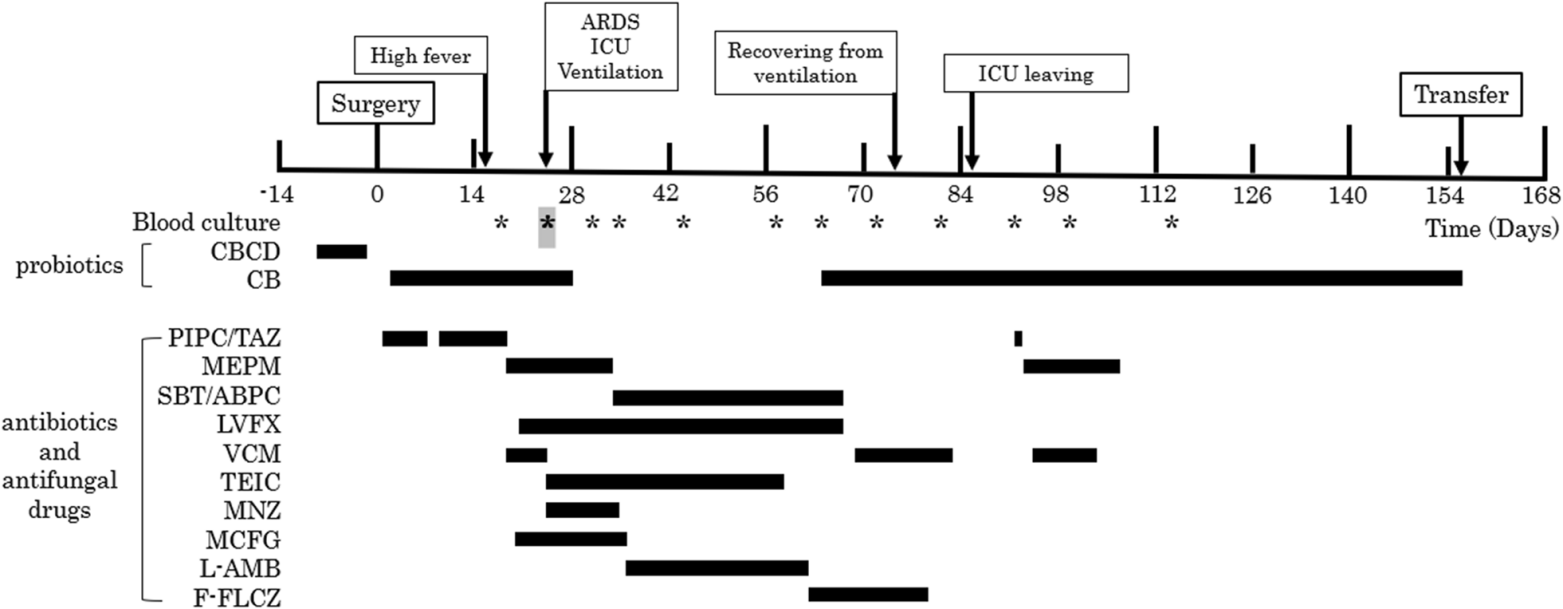
Postoperative course of case 2. Asterisks indicate blood culture tests. A bold asterisk highlighted in gray indicates positive blood culture of *Clostridium butyricum*. ARDS, acute respiratory distress syndrome; *C. butyricum*, *Clostridium butyricum*; CBCD, *Clostridium butyricum* combined drug; F-FLCZ, fosfluconazole; ICU, intensive care unit; L-AMB, amphotericin B (liposomal); LVFX, levofloxacin; MCFG, micafungin; MEPM, meropenem; MNZ, metronidazole; PIPC/TAZ, piperacillin/tazobactam; SBT/ABPC, sulbactam/ampicillin; TEIC, teicoplanin; VCM, vancomycin hydrochloride

**Fig. 6:**
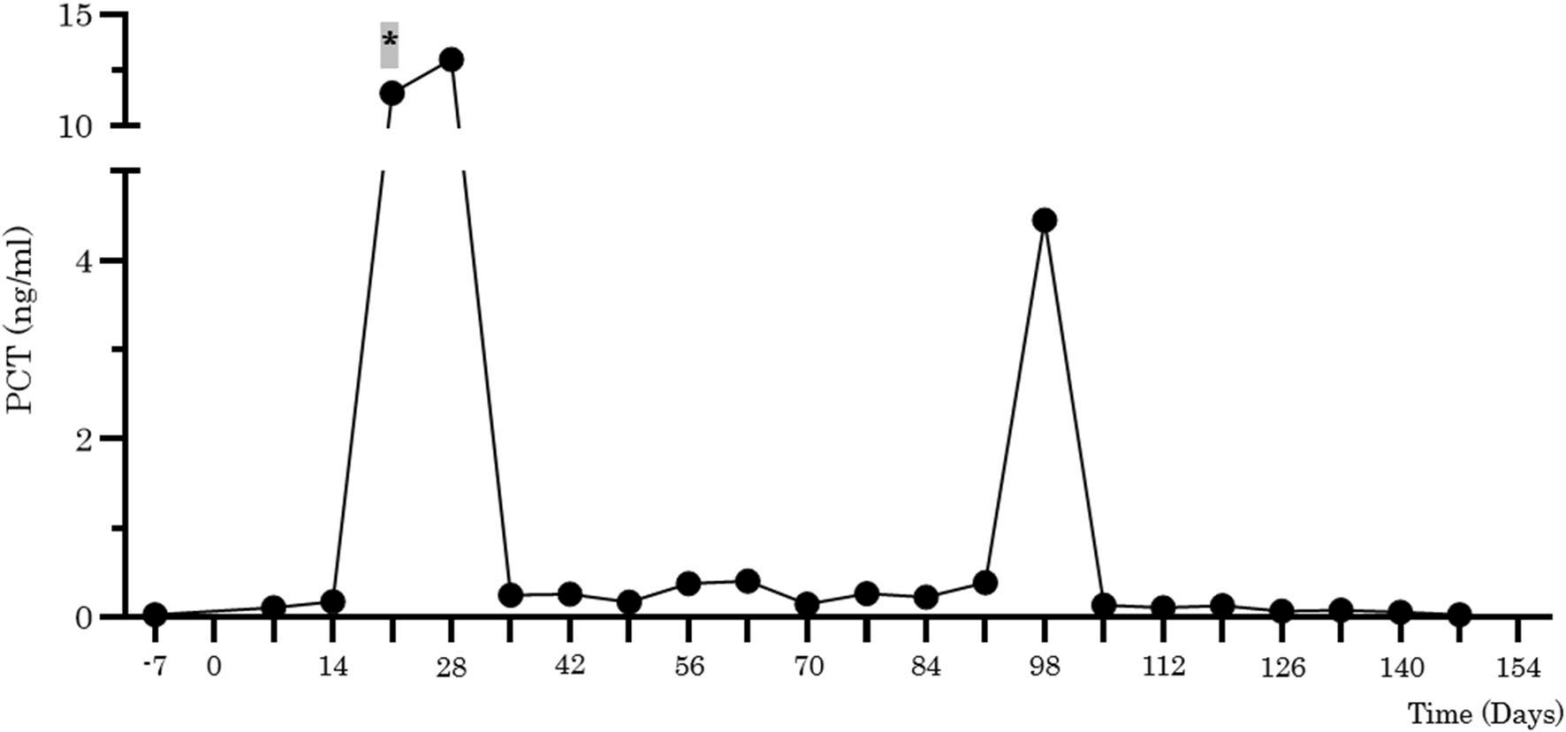
The change of serum procalcitonin value in case 2 during the perioperative period. A bold asterisk highlighted in gray indicates positive blood culture of *Clostridium butyricum*. PCT, procalcitonin

A 63-year-old man with diabetes mellitus visited a previous hospital due to a high-grade fever as the chief complaint. The patient had past histories of surgery: total colectomy for familial adenomatous polyposis at the age of 19, papillectomy with papilloplasty for a duodenal papillary tumor at the age of 34, and Frey’s operation for chronic pancreatitis at the age of 48. The patient was regularly taking combined probiotics, *Enterococcus faecium* T-110, *C. butyricum* TO-A, and *Bacillus subtilis* TO-A, before surgery. The patient was referred to our hospital for further examinations and treatment. Enhanced CT scan showed dilatation of the bile duct in the left liver and a mildly enhanced tumor in the dilatation origin (Fig. 4a). Magnetic resonance cholangio-pancreatgraphy (MRCP) revealed a defect from the common hepatic duct to the left hepatic duct (Fig. 4b). Biopsy of the tumor histologically demonstrated adenocarcinoma. The patient was diagnosed with perihilar cholangiocarcinoma. EBD in the left hepatic duct was performed to avoid repeated cholangitis. *Pseudomonas aeruginosa*, coagulase-negative *Staphylococcus* and a yeast-like fungus were observed in the preoperative nasopharyngeal screening cultures (Table 2). Preoperative culture of the bile juice showed various types of bacteria including *Pseudomonas aeruginosa* (Table 2). The preoperative blood test showed malnutrition (ALB 3.4 g/dl, pre-albumin 19.3 mg/dl, RBP 2.0 mg/dl), anemia (Hb 10.4 g/dl), and no jaundice (total-bilirubin 0.8 mg/dl). CRP level was within the normal range (0.01 mg/dl). T-Chol value was 127 mg/dl. The percentages of neutrophils and lymphocytes were 61.3% and 32.2%, respectively. Extended left hepatectomy with caudate lobectomy, extrahepatic bile duct resection and biliary reconstruction were performed. The operating time was 676 min, and the operative blood loss was 2396 ml. Histological examination demonstrated pTisN0M0. Surgical margin of the hilar bile duct was pathologically positive. Probiotic *C. butyricum* MIYAIRI 588 was started from postoperative day 1. The postoperative course went well without bile or anastomotic leakage until postoperative day 14. A high fever above 38 °C of unknown origin was observed day after day from postoperative day 15. *Klebsiella pneumonia* and *Enterococcus faecium* were identified from blood cultures on postoperative day 17 (Table 3). Meropenem (MEPM), levofloxacin (LVX) and vancomycin (VCM) were intravenously administered according to the drug sensitivity. However, the general condition became worse and progressed to respiratory failure, requiring mechanical ventilation at postoperative day 25 (Fig. 5). Blood cultures at postoperative day 25 revealed that *Enterococcus faecalis* and *C. butyricum* were positive in both of the two sets (Table 3), showing elevated serum PCT value (11.5 ng/ml) (Fig. 6). Probiotic-related bacteremia due to bacterial translocation was suspected as the infection route of *C. butyricum*. Thus, *C. butyricum* MIYAIRI 588 was stopped at postoperative day 27. Consequently, no *C. butyricum* was detected in the blood culture at postoperative day 30 (Table 3). This postoperative infectious complication improved with long-term intensive care. Although *C. butyricum* MIYAIRI 588 was administered at postoperative day 94 again, subsequent blood cultures showed no *C. butyricum*. The patient was transferred to another hospital for rehabilitation at postoperative day 156.

**Table 2:** Preoperative culture test of case 2

Sample	Detected bacteria
Throat swab	Normal flora
	*Pseudomonas aeruginosa*
	Yeast-like fungus
Nasal swab	Normal flora
	Coagulase (−) *Staphylococcus*
Bile	*Pseudomonas aeruginosa*
	*Klebsiella pneumoniae*
	*Escherichia coli*
	*Stenotrophomonas maltophilia*
	*Klebsiella oxytoca*
	*Candida albicans*

**Table 3:** Postoperative culture test of case 2

Collection date (postoperative day)	Sample	Detected bacteria
9	Bile	*Acinetobacter* sp. (MBL)
		*Enterobacter faecium*
		*Escherichia coli*
		*Klebsiella pneumoniae*
		*Pseudomonas aeruginosa*
		*Candida albicans*
12	Ascites	*Acinetobacter* sp. (MBL)
		*Enterobacter faecium*
		*Enterobacter faecalis*
		*Escherichia coli*
		*Klebsiella pneumoniae*
		*Candida albicans*
17	Blood	*Enterobacter faecium*^b^
		*Klebsiella pneumoniae*^b^
25	Blood	*Enterobacter faecalis*^b^
		*Clostridium butyricum*^b^
		*Enterobacter cloacae*
25	Stool	*Enterococcus* sp.
		*Enterobacter cloacae*
		*Bacillus subtilis*
		Coagulase (−) *Staphylococcus*
		*Candida albicans*
		*Candida parapsilosis*
30	Blood	*Candida albicans*^a^
34	Stool	*Enterococcus* sp.
		*Acinetobacter baumannii* complex
		*Candida albicans*
		*Candida parapsilosis*
36	Blood	Negative^b^
44	Blood	Negative^b^
51	Blood	*Candida parapsilosis*^a^
57	Blood	*Candida parapsilosis*^b^
		*Candida albicans*^a^
64	Blood	*Enterococcus faecium*^b^
72	Blood	Negative^b^
78	Urine	*Candida parapsilosis*
79	Blood	Negative^b^
83	Sputum	*Serratia marcescens*
91	Blood	*Serratia marcescens*^b^
96	Urine	Negative
99	Blood	*Staphylococcus epidermidis*^a^
114	Sputum	*Pseudomonas aeruginosa*
		*Enterococcus* sp.
		*Serratia marcescens*
114	Blood	Negative^b^

*MBL* metallo-β-lactamase

^a^Positive in one of two sets

^b^Positive in both of two sets

^c^One set examined

## Identifying colonies isolated from blood cultures in the two cases

Experiments to identify the colonies isolated from blood cultures in the two cases were conducted with the support of Miyarisan Pharmaceutical Co., Ltd. Samples at postoperative day 26 and 45 in case 1 and at postoperative day 25 in case 2 were examined. At first, 16S ribosomal RNA sequencing was performed. Then, sequence similarity was searched with Basic Local Alignment Search Tool (BLAST) of the National Center for Biotechnology Information (NCBI). From the sequencing analysis, all samples were identified as *C. butyricum*. Second, a cross-streak method using bacteriophage, phage KM1, was employed [10]. Positive and negative controls were *C. butyricum* MIYAIRI 588 and *C. butyricum* ATCC 19398^T^, respectively. In the result, none of the samples including the positive control grew in the phage zone, whereas the negative control did (data not shown). Therefore, the bacteria isolated from the blood cultures were very likely to be *C. butyricum* MIYAIRI 588 in all samples.

## Discussion

The present two cases showed bacteremia of *C. butyricum* during the treatment of sepsis after major hepatectomy for biliary cancer. Blood culture after stopping probiotic *C. butyricum* MIYAIRI 588 demonstrated no *C. butyricum* in both cases. Moreover, experiments to identify the bacteria isolated from the blood cultures revealed that they were very likely to be *C. butyricum* MIYAIRI 588 in both cases. Taken together, the present cases were diagnosed as probiotic-related bacteremia. Probiotic-related bacteremia is rare, even in a perioperative situation. In the last two decades, incidence of probiotic-related bacteremia after major hepatectomy for biliary cancer is 0.6% in our institute. Although probiotics are widely used during the perioperative period to prevent surgical infectious complications, probiotic-related bacteremia should be noted as one of the adverse events from the use of probiotics.

Boyle et al. have proposed risk factors for probiotics-related sepsis as follows: major risk factors are compromised immunity and premature infants. Minor risk factors were central venous catheter, impaired intestinal epithelial barrier, administration of probiotics by jejunostomy, concomitant administration of broad-spectrum antibiotics to which probiotics are resistant, probiotics with properties of high mucosal adhesion or known pathogenicity and cardiac vascular disease [8]. Our two cases were debilitated due to major hepatectomy and subsequent severe sepsis. Moreover, they had a damaged intestinal epithelial barrier and concomitant administration of broad-spectrum antibiotics. Hence, these situations are thought to have caused bacterial translocation, leading to probiotic-related bacteremia.

Probiotics-related bacteremia is a rare adverse event. A systematic review revealed that in five cases (0.3%) of 1530 cancer patients in 17 studies there were probiotic-related bacteremia/fungemia/positive blood cultures [11]. *Lactobacillus* species, especially *Lactobacillus rhamnosus*, have been reported to be the most common pathogen among probiotic-related bacteria [8, 12, 13]. A PubMed search by the keyword of “*Clostridium butyricum*” and “bacteremia” or “sepsis” revealed no bacteremia emerging from probiotic *C. butyricum*. The present cases are the first report of bacteremia of probiotic *C. butyricum*. *C. butyricum* is a kind of indigenous bacteria isolated from 10 to 20% of human feces [14]. Therefore, in cases with positive blood culture of *C. butyricum* during the administration of probiotics that include it, it is necessary to consider indigenous bacteria as the origin in addition to the use of probiotics. Further examinations, such as sequencing analysis and a cross-streak method as in the present cases, are necessary for the determination of probiotic-related bacteremia.

In the present cases, bacterial translocation was inferred to have led to probiotic-related bacteremia. In addition to *C. butyricum*, other microorganisms were simultaneously identified in the blood cultures. *C. butyricum* may not be pathogenic in the bloodstream because of obligate anaerobes. The previous report, which demonstrated that *C. butyricum* MIYAIRI 588 has no genes for *Clostridium* toxins, supports the safety of *C. butyricum* MIYAIRI 588 from molecular biological assessment [15]. Also, *C. butyricum* MIYAIRI 588 has been reported to be susceptible to commonly used antibiotics, including PIPC/TAZ, VCM, MEPM and LVX, which were administered in the present cases [15, 16]. Therefore, if *C. butyricum* isolated from the blood of the present cases is *C. butyricum* MIYAIRI 588, it is presumed to have been in the state of spores, which are resistant to antibiotics and have an extremely low possibility of being pathogenic. Taken together, *C. butyricum* MIYAIRI 588 may not have pathogenicity in cases of probiotic-related bacteremia that occur during the administration of sensitive antibiotics. However, the state of probiotic-related bacteremia may indicate the likelihood of passage of other pathogenic bacteria across the intestinal epithelium into the bloodstream, namely, the likelihood of bacterial translocation. On the other hand, various reports have shown that probiotics are beneficial for bacterial translocation [17–19]. Thus, we should not be excessively reluctant to use probiotics because of the possibility of bacterial translocation, while being aware of probiotic-related bacteremia.

There is a limitation in the cross-streak method using phage KM1 performed in the present cases. That is, there may be bacteria other than *C. butyricum* MIYAIRI 588 that are sensitive to phage KM1.

## Conclusion

We experienced two rare cases of probiotic-related bacteremia after major hepatectomy for biliary cancer. Probiotic-related bacteremia should be noted as one of the possible adverse events when using probiotics.

## Data Availability

The datasets supporting the conclusions of this article are included within the article.

## Abbreviations

ABPC: Ampicillin
ARDS: Acute respiratory distress syndrome
ARLAB: Antibiotic-resistant lactic acid bacteria
*C. butyricum*: *Clostridium butyricum*
CBCD: *Clostridium butyricum* combined drug
CMZ: Cefmetazole
CRP: C-reactive protein
CT: Computed tomography
DAP: Daptomycin
DIC: Disseminated intravascular coagulation
F-FLCZ: Fosfluconazole
ICG: Indocyanine green
ICU: Intensive care unit
L-AMB: Amphotericin B (liposomal)
LVX: Levofloxacin
MCFG: Micafungin
MEPM: Meropenem
MNZ: Metronidazole
NAC: Neoadjuvant chemotherapy
PCT: Procalcitonin
PIPC/TAZ: Piperacillin/tazobactam
RBP: Retinol-binding protein
RHA: Right hepatic artery
SBT/CPZ: Sulbactam/cefoperazone; sulbactam/ampicillin
SBT/ABPC: Sulbactam/ampicillin
T-Chol: Total cholesterol
TEIC: Teicoplanin
VCM: Vancomycin hydrochloride
